# The H3K27me3 histone mark correlates with repression of colour and aroma development post-harvest in strawberry fruit

**DOI:** 10.1093/jxb/erae464

**Published:** 2024-11-15

**Authors:** Ashley Baldwin, Tamara Lechon, Angela Marchbank, Simon Scofield, Kerstin Lieu, Charlotte L Wilson, Richard A Ludlow, Robert J Herbert, Hans-Wilhelm Nützmann, Hilary J Rogers

**Affiliations:** School of Biosciences, Cardiff University, Sir Martin Evans Building, Museum Avenue, Cardiff CF10 3AX, UK; School of Biosciences, Cardiff University, Sir Martin Evans Building, Museum Avenue, Cardiff CF10 3AX, UK; School of Biosciences, Cardiff University, Sir Martin Evans Building, Museum Avenue, Cardiff CF10 3AX, UK; School of Biosciences, Cardiff University, Sir Martin Evans Building, Museum Avenue, Cardiff CF10 3AX, UK; School of Biosciences, Cardiff University, Sir Martin Evans Building, Museum Avenue, Cardiff CF10 3AX, UK; School of Biosciences, Cardiff University, Sir Martin Evans Building, Museum Avenue, Cardiff CF10 3AX, UK; School of Biosciences, Cardiff University, Sir Martin Evans Building, Museum Avenue, Cardiff CF10 3AX, UK; School of Biosciences, Cardiff University, Sir Martin Evans Building, Museum Avenue, Cardiff CF10 3AX, UK; School of Science and the Environment, University of Worcester, Henwick Grove, Worcester WR2 6AJ, UK; Biosciences, University of Exeter, Geoffrey Pope Building, Stocker Road, Exeter EX4 4QD, UK; School of Biosciences, Cardiff University, Sir Martin Evans Building, Museum Avenue, Cardiff CF10 3AX, UK; University of Padova, Italy

**Keywords:** ChIP-seq, *Fragaria×ananassa*, fruit, H3K27me3, histone methylation, post-harvest, RNA-seq

## Abstract

Strawberry ripening is non-climacteric, and post-harvest fruit enter senescence and deteriorate rapidly. Chilled storage induces transcriptome-wide changes in gene expression, including down-regulation of aroma-related genes. Histone marks are associated with transcriptional activation or repression; the H3K27me3 mark is mainly associated with repression of gene expression. Here genes associated with H3K27me3 were identified through ChIP-seq in ripe red strawberry fruit at harvest and after 5 d of chilled storage in the dark. The number of ChIP peaks increased with storage time, indicating an increased role for this mark in regulation of gene expression following chilled dark storage. Comparing ChIP-seq data with RNA-seq data from the same material identified 440 genes whose expression correlated with H3K27me3 repression. Abiotic stress genes, especially cold stress response genes, were down-regulated during storage. Increased association with the H3K27me3 mark indicated that they may be repressed via this epigenetic mark. Other functional groups included cell wall and carbohydrate metabolism. The association with the H3K27me3 mark of two transcription factor genes (*FaHY5* and *FaTRAB1*) and of *FaADH*, involved in ester biosynthesis, was validated by ChIP-PCR. These three genes were all down-regulated during storage and indicated a network of H3K27me3 gene repression affecting both anthocyanin and ester biosynthesis.

## Teaser

A correlation is presented between the H3K27me3 histone mark and repression of a transcription factor cascade regulating colour and aroma development during dark chilled post-harvest storage of strawberry fruit.

## Introduction

Strawberry fruit develop from a single inflorescence as an aggregate fruit, where the receptacle comprises the main edible part of the fruit. This process involves structural, physiological, and biochemical changes (Moya-León *et al.*, 2019), resulting in an increase in sugar content, a change in fruit colour from green to red, as well as secondary metabolite and aroma biosynthesis (Kim *et al.*, 2013; Moya-León *et al.*, 2019; Sánchez-Gómez *et al.*, 2022). Strawberries are non-climacteric (Gu *et al.*, 2019), therefore they do not increase their respiratory rate and are not responsive to ethylene post-harvest (Jia *et al.*, 2011). Consequently, complete ripening occurs only on the plant, and strawberry fruit are picked close to full maturity when they are in their final stages of ripening. Post-harvest, fruit initiates fruit senescence involving macromolecule breakdown and tissue softening, and ending in cell death (Yun *et al.*, 2012; Chen *et al.*, 2014). As a result of this, they are highly perishable as they are less firm at harvest than climacteric fruit, increasing their susceptibility to mechanical damage and spoilage microorganisms (Siebeneichler *et al.*, 2020). Post-harvest senescence is accelerated by respiration which is increased in wounded fruit, as well as by dehydration (Yang *et al.*, 2014). Dehydration post-harvest also up-regulates abscisic acid (ABA) signalling (Chen *et al.*, 2014, 2016). This in turn promotes anthocyanin biosynthesis (Chen *et al.*, 2016). During strawberry ripening, this occurs through the activation of a transcription factor network involving TRAB1, MADS1 (Lu *et al.*, 2023), HY5, RIF (Li *et al.*, 2020; Li *et al*., 2023; Liu *et al.*, 2023), and MYB10 (Medina-Puche *et al.*, 2014). HY5 has also been shown to regulate post-harvest anthocyanin biosynthesis (Liu *et al.*, 2023). MADS1 has a role in repressing several aspects of strawberry fruit ripening. It represses several enzymes in the anthocyanin biosynthesis pathway as well as enzymes involved in cell wall modulation leading to softening, and the biosynthesis of important components of the strawberry aroma profile (Lu *et al.*, 2023). Specifically, MADS1 represses the expression of AAT (alcohol acyl transferase). AAT is a key enzyme in ester biosynthesis, whose expression is down-regulated during chilled post-harvest storage of strawberry fruit (Baldwin *et al.*, 2023). This correlates with changes in the ester profile of the aroma. Esters are the major components of the strawberry aroma, and during 5 d of post-harvest storage at 8 °C the relative abundance of non-acetate esters in the aroma profile drops significantly (Baldwin *et al.*, 2023).

Strawberry fruit have a short shelf-life post-harvest, especially when used in ready to eat fresh fruit salads, where they are also often halved. Hence, in the commercial supply chain, chilling is used to delay post-ripening senescence and reduce post-harvest spoilage from resident and opportunistic microflora (Feliziani and Romanazzi, 2016). Cold dark storage results in a transcriptomic and metabolic shift (Baldwin *et al.*, 2023), down-regulating >1000 genes including those encoding numerous transcription factors and genes related to aroma biosynthesis. This shift in metabolism as a result of cold storage has also been reported in other soft fruit such as tomato (Zhang *et al.*, 2016) where a down-regulation in the expression of flavour-related genes was ascribed to promoter methylation. In *Fragaria vesca*, DNA methylase and demethylase are highly expressed during fruit ripening, thus suggesting an involvement in regulation of gene expression (Gu *et al.*, 2016). DNA methylation is also involved in regulation of senescence in other plant organs such as leaves (Yuan *et al.*, 2020).

Other forms of epigenetic control have also been associated with plant organ senescence, including histone methylation (Cigliano *et al*., 2022; Zhang *et al*., 2022). Histone methylation can act to either repress or activate genes: for example, deposition of H3K4me3 and H3K36me3 marks is often observed on actively expressed genes, whereas H3K27me3 is often observed on repressed genes (Liu *et al*., 2010). In tomato, genome-wide studies estimated that about half of the genes differentially expressed during post-harvest storage were epigenetically regulated, with a reduction in genes associated with the repressive histone mark H3K27me3 (Cigliano *et al*., 2022). H3K27me3 has also been shown to regulate ester formation in peach and apple, with H3K27me3 removal from NAC transcription factor genes and aroma-related genes such as *AAT* increasing their expression during ripening (Cao *et al*., 2021). In strawberry, the genome-wide distribution of H3K9/K14 acetylation in the red-stage strawberry receptacles and H3K27me3 in red-stage fruits was used to characterize transcriptional regulation of expansin genes by histone modifications (Huang *et al*., 2020; Mu *et al*., 2021). Transcript levels of expansin genes were correlated with both histone marks, suggesting that they are under epigenetic control in strawberry fruit. Interestingly, while H3K27me3 marks are typically enriched across the whole gene body (Liu *et al*., 2010; Payá-Milans *et al*., 2019; Cigliano *et al*., 2022), Mu *et al*. (2021) showed H3K27me3 enrichment primarily at the 5' ends and promoters of two expansin genes in *F. vesca*.

Given the importance of epigenetic control both during senescence and post-harvest, the regulation of expression by the H3K27me3 mark was explored here in halved strawberries during a 5 d storage period where fruit were exposed to wounding followed by dark cold conditions. Using a genome-wide ChIP-seq approach with a commercial strawberry cultivar, we show that a large number of genes are indeed associated with both loss and gain of the H3K27me3 mark. Furthermore, our study suggests that this epigenetic regulator is involved in controlling both colour development and aroma production post-harvest in strawberries.

## Materials and methods

### Plant material

*Fragaria×ananassa* cv. Elsanta plants were greenhouse grown at a minimum temperature of 19 °C with supplementary lighting (16 h). Harvested fruit were homogeneous in size and colour (at the red ripe stage) with no external damage. They were immersed in 100 ml of 200 ppm sodium hypochlorite for 2 min, and air dried. The calyx was removed and fruits were halved: one half was processed immediately and the other was stored for 5 d at 8 °C in a Jeiotech IL-21chilled incubator in the dark. Three replicates of three half fruits each were used per time point. Fruit were then skinned rapidly using a razor blade to remove all seeds, and snap-frozen in liquid nitrogen.

### RNA-seq

RNA samples were extracted from fruit tissue (described above) following the protocol of Greco *et al.* (2014) and adapted by Dhorajiwala *et al.* (2021). RNA quality and concentration were verified by agarose gel electrophoresis, a Nanodrop spectrophotometer (Thermo Scientific), and an Agilent 2100 Bioanalyzer (Agilent). RNA was used for library preparation [poly(A) enrichment], tested using a Qubit 2.0 and an Agilent 2100 for concentration and insert size. Sequencing used an Illumina NovaSeq 6000, generating ≥40 million paired-end reads per sample. Library preparation and sequencing was performed by Novogene Co., Ltd (Beijing, China). Sequences were quality checked and trimmed in house by Novogene Co., Ltd and then aligned to the *F. × ananassa* ‘Royal Royce’ Genome v1.0 (Hardigan *et al.*, 2021, Preprint) using HISAT2 v. 2.0.5 (Kim *et al.*, 2019). A list of differentially expressed genes (DEGs) (*P*-adj <0.05, log_2_-fold change >1 and < –1) was generated via DESeq2 (Love *et al.*, 2014). For downstream analyses, the transcriptome was compared with the *F. vesca* Whole Genome v2.0.a1 Assembly & Annotation (Tennessen *et al.*, 2014) via BLASTX.

### Nuclear isolation, DNA fragmentation, and chromatin immunoprecipitation

Isolation of nuclei, DNA fragmentation, and ChIP were according to Huang *et al.* (2020) starting with 2 g of frozen powdered fruit material. Nuclei were isolated by homogenization using a glass homogenizer (Kimble Chase, Ficher Scientific). Chromatin fragmentation used 30 U of micrococcal nuclease (ThermoScientific, # EN0181) digestion optimized to a 10 min incubation time. ChIP was performed with native chromatin: Dynabeads Protein A (Thermo Fisher, cat. No 10001D) were used to pre-clear the digested chromatin, and a H3K27me3 antibody (Sigma Aldrich, # 07-449) bound to Dynabeads was used to immunoprecipitate bound chromatin. DNA was recovered from the chromatin using a Monarch® PCR & DNA Cleanup Kit (New England Biolabs, #T1030S) alongside an input sample, namely fragmented chromatin that was not immunoprecipitated. Three biological replicates were used for each time point (day 0 and day 5 of chilled dark storage).

### ChIP-seq

Immunoprecipitated DNA concentration was checked using a Qubit Fluorometer, and DNA quality via a High Sensitivity D1000 ScreenTape (Agilent, #5067-5584). A NEXTflex™ Rapid DNA-Seq Kit (PerkinElmer # NOVA-5188) was used for paired-end DNA library construction. Sequencing used an Illumina® Next Seq 5000 Platform (120 million reads per run) performed by the Biosciences Genomics Research Hub within the School of Biosciences, Cardiff University, UK.

Low quality reads and sequencing adaptors were removed using Fastp (v0.20, Chen *et al.*, 2018) and quality checked using Fastqc (v0.11.9) and MultiQC (v1.9, Ewels *et al.*, 2016). Trimmed FASTQ reads were mapped using Bowtie2 (v2.4.1, Langmead and Salzberg 2012) employing a maximum fragment length of 500 and the forward–reverse option to the three available genomes: *F. × ananassa* ‘Royal Royce’ Genome v1.0 (https://www.rosaceae.org/Analysis/12335030, Hardigan *et al.*, 2021, Preprint), *F. × ananassa* ‘Camarosa’ Genome Assembly v1.0.a1 (https://www.rosaceae.org/species/fragaria_x_ananassa/genome_v1.0.a1, Edger *et al.*, 2019), and *F. vesca* whole genome v2.0.a1 (https://www.rosaceae.org/species/fragaria_vesca/genome_v2.0.a1, Tennessen *et al.*, 2014). The resulting Sequence Alignment/Map (SAM) format files were converted into Binary Alignment Map (BAM) format and sorted by chromosome using Samtools (v1.10). Using Samtools, Bamtools (v2.5.1), and Deeptools (v3.3.0), bigwig files (binSize=50) were created to load into IGV (v2.12.3) to assess coverage. Peak calling was performed using MACS2 (v2.2.4, Zhang *et al.*, 2008) with the broad calling argument (--broad -p 1e-3 -g 786543374). ChIPQC (v4.3, Carroll *et al.*, 2014 within R v4.1.1) was used to assess sample quality. Following this analysis, the two replicates with the highest quality for each time point were merged (Supplementary Fig. S1), and the lowest quality replicate was excluded from further analyses. This improved the quality scores: determined by the SSD and percentage of reads in peaks (RiP%) (Supplementary Table S1). Pseudo-replicates were created for both pull-down and input samples. Irreproducibility Discovery Rate (IDR v2.0.3) analysis using pseudo-replicate peak calls as well as non-pseudo-replicate peak calls was conducted to assess replicability. BEDTools (v2.29.2, Quinlan and Hall, 2010) intersect was used to find common peaks across pseudo-replicates. Coverage, peak length, and the peak position were assessed using IGV. Diffbind (v3.17, Stark and Brown, 2011) was used to look for differences between storage time points (day 0 and day 5). ChIPseeker (v3.17, Yu *et al.*, 2015) was used for peak annotation and peak exploration. Gene ontology (GO) term enrichment was performed using PlantRegMap (Tian *et al.*, 2020) and visualized using the R package GOplot (Walter *et al.*, 2015). IGV was used to visually assess the peak quality. The Kyoto Encyclopedia of Genes and Genomes (KEGG) was also used to compare pathway changes between day 0 and day 5 of cold dark storage.

### ChIP-qPCR and RNA real-time quantitative PCR

Total RNA extracted as above was DNase treated and cDNA was synthesized using the GoScript Reverse Transcription Mix, Oligo(dT) (Promega). Primers were designed using primer3 (http://primer3.ut.ee/; Rozen and Skaletsky 2000) and are listed in Supplementary Table S2. The real-time quantitative PCR (RT-qPCR) was conducted using a LightCycler® 96 instrument (Roche) and SyGreen Blue Mix Lo-ROX (PCR Biosystems Ltd, London, UK). Relative gene expression was quantified using the delta-delta Ct (2^DDCt) method (Livak and Schmittgen, 2001) using the gene encoding the small ribosomal subunit 40S as a reference gene (stable expression validated from the transcriptome data). For the ChIP validation, the percentage input method was used to calculate enrichment using 18S for validation (Supplementary Fig. S2A). All PCRs were conducted using three biological replicates and two technical replicates.

### Analysis of total anthocyanins

Strawberry fruit (*F. × ananassa* cv. Elsanta) were harvested at the red ripe stage and subjected to 5 d either at 8 °C in the dark in a Jeiotech IL-21chilled incubator or at 20 °C in the light (150 µmol m^–2^ s^–1^). Fruit were processed as described above, and 0.2 g of material, ground under liquid nitrogen, was used for spectrophotometric analysis of total anthocyanins according to Klopotek *et al.* (2005), expressing the values as µg of pelargonidin-3-glucoside g^–1^ FW.

### Statistical analyses

Depending on whether the data were normally distributed, either a one-way ANOVA with post-hoc Tukey test, or a Kruskal–Wallis statistical test with post-hoc Wilcoxon rank-sum was used to find statistical differences in gene expression between treatments. *P*-values of <0.05 were considered significant.

## Results

To better understand the effects of chilled storage on strawberry fruit gene expression, genes associated with the H3K27me3 mark post-harvest were identified. Halved fruit, typically used in prepared fruit salads, were assessed at harvest and after 5 d storage at 8 °C. Seeds were removed to focus on the changes in the fleshy receptacle.

### H3K27me3 profile changes during strawberry fruit storage

Prior to sequencing, the effectiveness of the H3K27me3 ChIP pull-down was tested by RT- qPCR for DNA extracted from fruit both at day 0 (at harvest) and day 5 (following 5 d of chilled dark storage) samples. Two genes were selected, based on their regulation by H3K27me3 in Arabidopsis (Zhang *et al.*, 2007). Orthologous genes were identified in the *F. vesca* genome and ranked on sequence identity. To ensure the selected genes were expressed in the fruit, the strawberry eFP browser (Hawkins *et al.*, 2017) was used to identify the top 10 genes that had low expression in fruit at the red ripe stage and high expression in other tissues. The genes selected were: strawberry gene Fxa4Ag102286 which is annotated as WRKY75 and Fxa7Ag202455 which is annotated as LOB domain-containing protein. The ChIP-qPCR data showed successful pull-down of Fxa4Ag102286 and Fxa7Ag202455, with both being >10% enriched in the H3K27me3 immunoprecipitation compared with the input at both time points (Supplementary Fig. S2A), while the control, 18S rRNA, was not enriched. The subsequent ChIP-seq and RNA-seq data confirmed that H3K27me3 peaks were associated with both these genes but there were very few RNA-seq reads, thus suggesting they have been silenced at both time points (Supplementary Fig. S2B).

Overall, between 46 036 568 and 77 704 802 reads were obtained for each sample from the ChIP-seq, comprising fruit from *F. × ananassa* cv. Elsanta at harvest and after 5 d of chilled dark storage. On average, 31 570 868 for the H3K27me3 ChIP pull-down and 57 844 169 for the input samples were mapped to the *F. × ananassa* cv. Royal Royce genome. Reads were also mapped to two other available genomes *F. × ananassa* cv. Camarosa and *F. vesca*, but the cv. Royal Royce provided the best percentage mapped reads (Supplementary Table S3). An inspection of the GC content revealed the presence of some bacterial DNA (Supplementary Fig. S3); these sequences are unlikely to have mapped onto the genome and therefore would not interfere with further analyses.

**Fig. 1.**
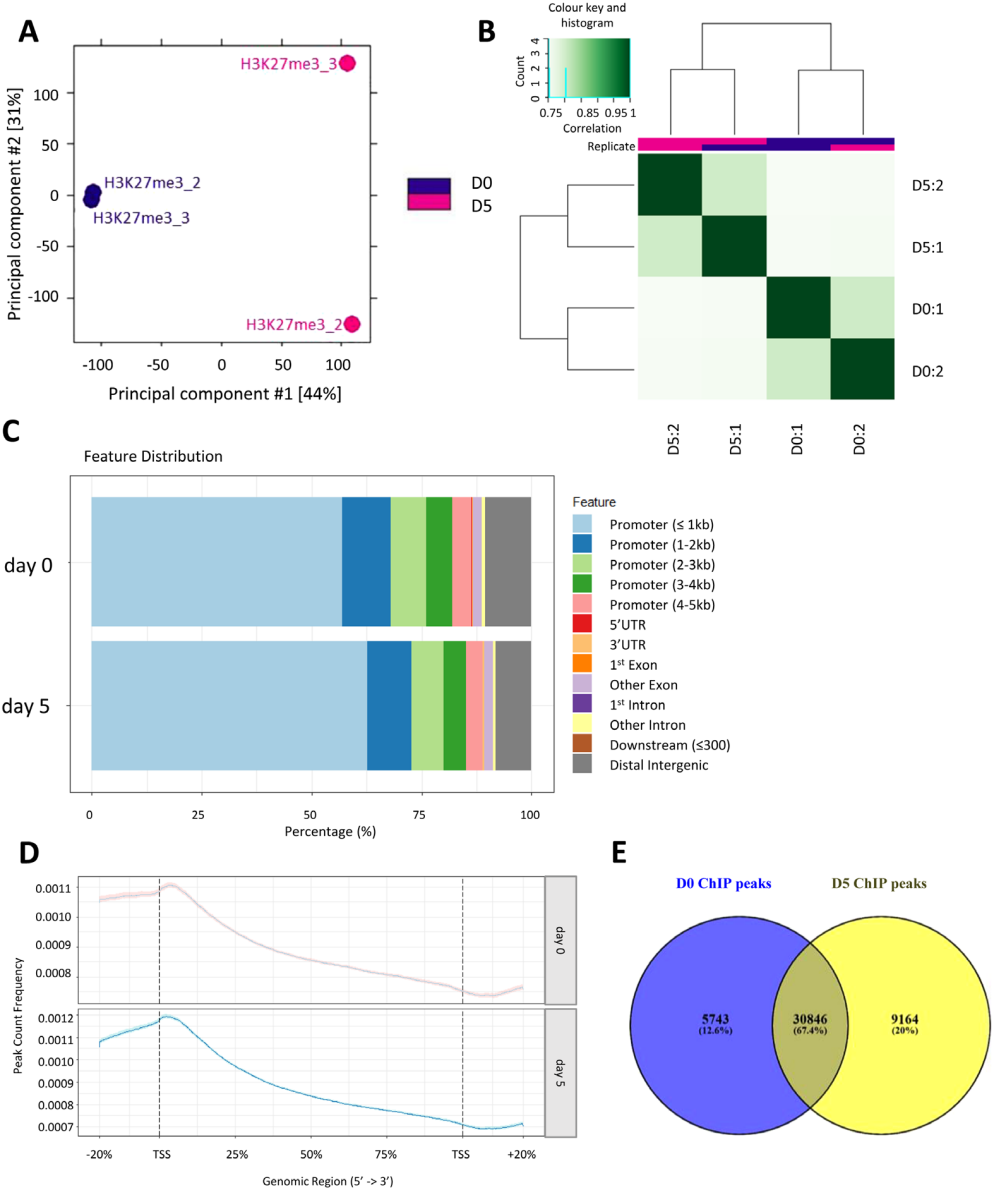
ChIP-seq analysis of the H3K27me3 profile across the *Fragaria*×*ananassa* genome comparing fruit at day 0 and day 5 of chilled dark storage. (A) Principal component analysis plot showing pseudo-replicate grouping by day of storage using all read counts. (B) Correlation heatmap, using H3K27me3 occupancy (peak caller score) data. (C) Genomic annotation of H3K27me3 for day 0 and day 5. Bar charts show the percentage of mapped peaks that were located in each genomic region. Promoter regions are divided into different portions; kilobases in parentheses are distances from the TSS. (D) Profile of H3K27me3 ChIP peak frequency across gene body regions (TSS, transcription start site; TTS transcriptional termination site). Mean profiles of ChIP peaks are shown from each time point, indicating their location on the gene and the peak count frequency for that location. (E) Overlap between the annotated ChIP peaks from day 0 and day 5 stored strawberry fruit.

Principal component analysis grouped the pseudo-replicates by day of storage (Fig. 1A) and they showed good grouping in a correlation heatmap (Fig. 1B). The ChIP-seq peaks largely occurred within the promoter region and were less frequent in the gene body [5'-untranslated regions (UTRs), exons, introns, and 3' UTRs] (Fig. 1C). Some peaks occurred in the distal intergenic region: the genomic region located between two genes or gene loci but relatively distant from both. The peaks also seemed to decrease across the gene body, dropping just after the transcription start site (TSS) (Fig. 1D). The median peak length for day 0 was 1045 bp and for day 5 was 922 bp. The average peak length was 1966 bp for day 0 and 1783 bp for day 5.

A total of 45 753 H3K27me3 ChIP peaks were annotated across the two time points of strawberry fruit storage, with the majority shared between day 0 and day 5 (Fig. 1E). However, >5000 peaks (12.6%) were unique to day 0 and >9000 peaks (20%) were unique to day 5 fruit, indicating that the pattern of H3K27me3 binding changes during storage. The strawberry genome contains 101 793 protein-coding genes (*F. × ananassa* Royal Royce v1.0), thus in total day 0 peaks cover ~36% of the total gene number and day 5 peaks cover ~39%.

### Over 400 genes are potentially down-regulated by H3K27me3 modification

To assess whether H3K27me3 modifications might be correlated with gene expression during fruit storage, the annotated H3K27me3 ChIP peaks were compared with DEGs from a transcriptomic analysis of the same fruit material. Over 75% of the RNA-seq reads mapped to the ‘Royal Royce’ reference genome (Supplementary Table S4), with an average of >86 million reads for both day 0 and day 5. A total of 62 626 genes were expressed at day 0, and 63 840 genes at day 5 of storage. By HISAT2 analysis, 6382 DEGs (*P*-adj <0.05, log_2_-fold change >1) were identified between day 0 and day 5 of cold dark storage, with 2292 down-regulated and 4090 up-regulated (Supplementary Fig. S4A). GO annotation of all DEGs showed defence response as the most significantly changed biological process, and extracellular region and cell wall as the most enriched components (Supplementary Fig. S4B). KEGG enrichment showed that phenylpropanoid biosynthesis was the most enriched metabolic pathway (Supplementary Fig. S4C). Other pathways that were significantly enriched include mitogen-activated protein kinase (MAPK) signalling, starch and sucrose metabolism, and flavonoid biosynthesis (Supplementary Table S5).

A comparison of the annotated RNA-seq and ChIP-seq peaks (Supplementary Fig. S5) revealed 41 325 genes expressed at day 0, and 37 493 genes expressed at day 5 that were not associated with the H3K27me3 mark on each of those days. The majority of expressed genes at day 0 and day 5 were not associated with H3K27me3, as expected given the repressive role of H3K27me3. However, a further 21 301 genes on day 0 and 26 347 on day 5 were both expressed and associated with ChIP peaks. Of those ChIP peaks that were not associated with expressed genes at each time point, 11 744 were shared. GO term annotation of these repressed genes (Supplementary Table S6) is broadly consistent with functions expected to be repressed in fruit, including flower and shoot system development, and several biosynthetic processes.

**Fig. 2.**
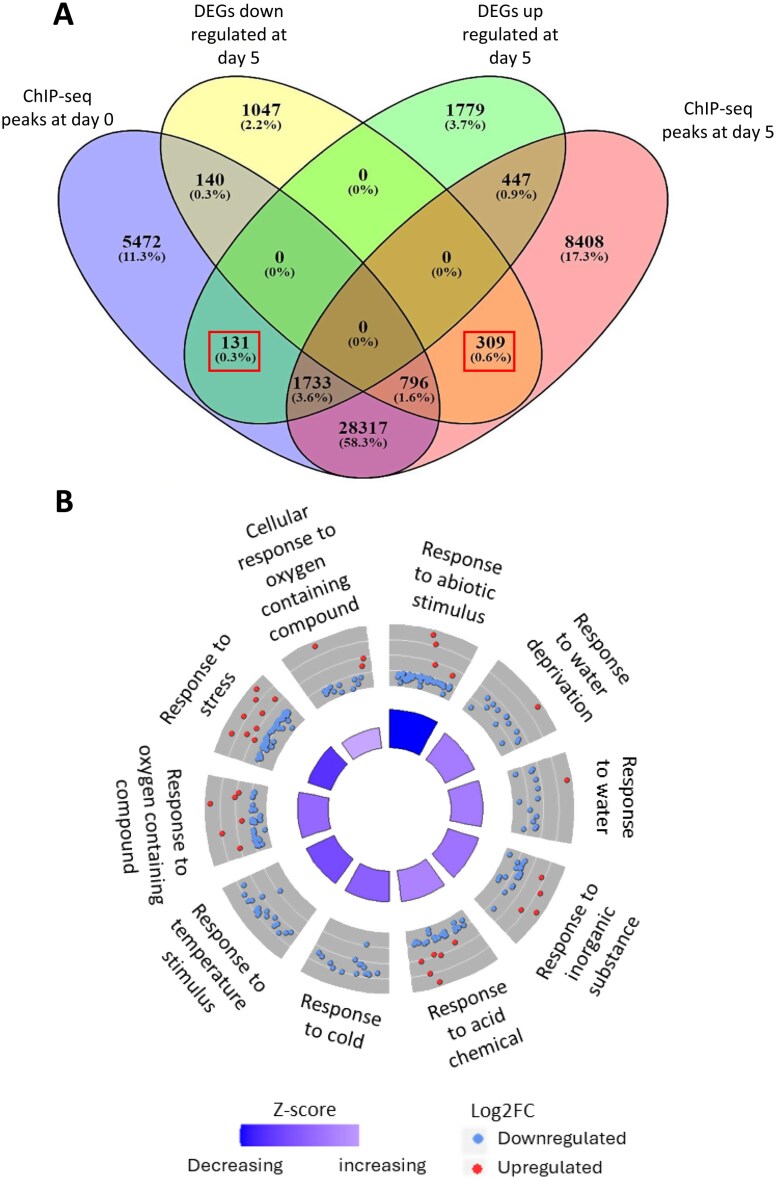
Comparison of H3K27me3 ChIP-seq and RNAseq datasets for stored strawberry fruit. (A) Overlap in expression patterns of RNAseq DEGs (up- and down-regulated) and ChIP-seq peaks at day 0 and day 5 of chilled storage. (B) GO term plot of the most significant GO terms (BP) in the 440 genes whose expression change between day 0 and day 5 of storage is consistent with H3K27me3 regulation. The outer circle shows a scatter plot for each term of the log_2_-fold change of the assigned genes. Red circles display up-regulation and blue down-regulation. The inner circle shows the significance of the GO term, with a taller segment representing higher significance. Inner circle colour represents the GO term *Z*-score. This reports the proportion of up- (increasing) and down- (decreasing) regulated genes during storage represented in the GO term, compared with the set of 440 genes.

Comparison of RNA-seq DEGs and ChIP-seq peaks (Fig. 2A) included 131 genes that were up-regulated at day 5 compared with day 0 in the RNA-seq analysis and corresponded to ChIP-seq peaks at day 0 but not at day 5. Conversely, 309 genes which were down-regulated at day 5 were associated with ChIP-seq peaks at day 5 but not day 0 (Supplementary Table S7). The pattern of expression change and ChIP-seq peaks for these genes is consistent with a potential repression of their gene expression during storage by H3K27me3 binding. The position of the histone mark is also consistent with their regulation by H3K27me3: 203 were found within the promoter region (–2000 bp from the TSS) and 362 were found within –2000 bp to +2000 bp from the TSS. However, a significant number of the DEGs were not associated with ChIP-seq peaks, and vice versa, and a further 2529 DEGs (1733 up-regulated and 796 down-regulated) showed an unexpected positive correlation between ChIP-seq peaks and gene expression levels.

GO term enrichment showed that many of the 440 genes whose expression and ChIP peaks negatively correlate during storage, consistent with them being repressed by H3K27me3, were associated with temperature, cold, water deprivation, and water (Fig. 2B). The genes whose expression was up-regulated during storage were more represented in the enriched GO terms: ‘response to stress’, ‘response to abiotic stimulus’, and responses to oxygen-containing compounds and acids. The most significant term was ‘response to abiotic stimulus’, and this was also the term that included the highest proportion of the down-regulated genes amongst the group of 440 genes.

Six GO terms were also shared between the whole RNA-seq dataset and the 440 genes likely to be H3K27me3 regulated. Three of these: GO:0071944, ‘cell periphery’; GO:0016759, ‘cellulose synthase activity’; and GO:0016760, ‘cellulose synthase (UDP-forming) activity’ relate to cell wall metabolism. Of the 50 genes in these GO terms, the vast majority (41/50) were down-regulated in expression during storage. The other GO terms represented were: GO:0016872, ‘intramolecular lyase activity’; GO:0030246, ‘carbohydrate binding’; and GO:0030001, ‘metal ion transport’.

### H3K27me3-associated genes include transcription factor genes and metabolic genes

**Fig. 3.**
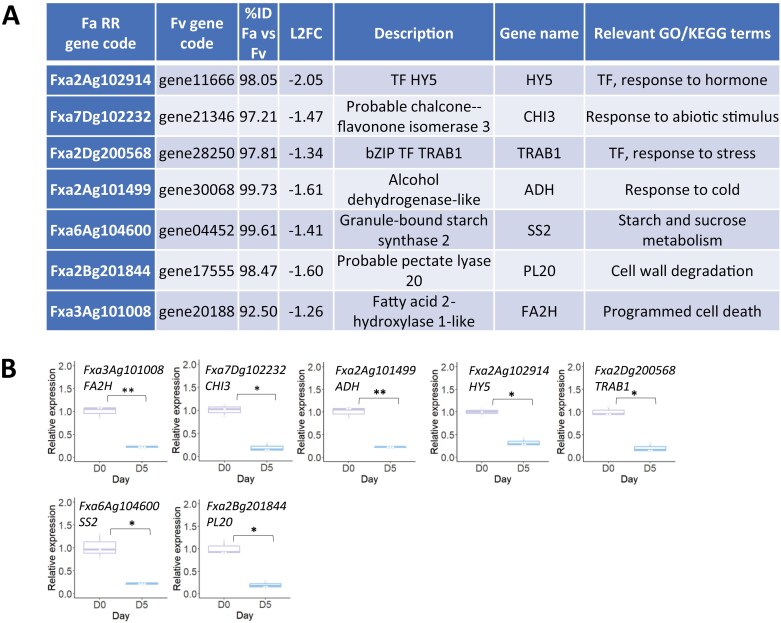
Seven genes from the ChIP-seq analysis selected for validation. (A) *Fragaria×ananassa* Royal Royce code, closest matched *Fragaria vesca* code, percentage identity between the two sequences, log_2_-fold change from the RNA-seq analysis, annotated function, gene name, and associated GO/KEGG terms. (B) Real-time qPCR analysis of the expression of these genes (mean ±SD) in fruit at day 0 and day 5 of chilled storage (*n*=3). Asterisks are based on a *t*-test; **P<0.01; *P<0.05; ns, non-significant.

Seven of these H3K27me3-associated genes were selected for validation of their expression during fruit storage (Fig. 3A). Genes were selected to represent functions and processes associated with fruit storage: general stress responses, cold stress response, cell death, and fruit quality, identified in the RNA-seq KEGG and GO enrichment analysis. The seven genes comprise two transcription factor genes (HY5 and TRAB1), one related to starch and sucrose metabolism (SS2), response to cold (ADH), flavonoid biosynthesis (CHI3), cell wall degradation (PL20), and cell death (FA2H). All seven genes were down-regulated between day 0 and day 5 of storage in agreement with the RNA-seq data (Fig. 3B).

**Fig. 4.**
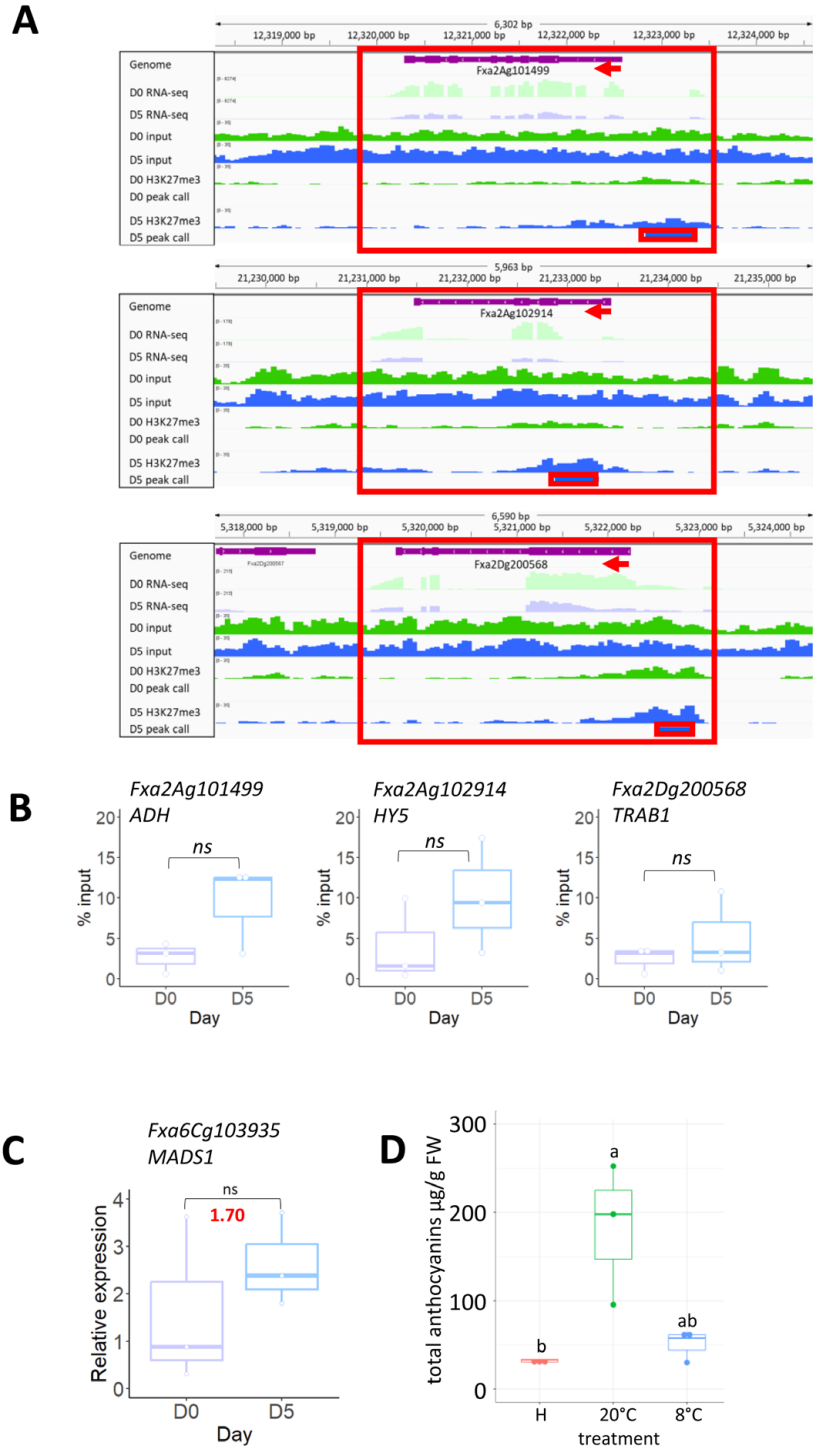
Validation of ChIP-seq data. (A) IGV tracks of RNA-seq data and ChIP-seq data. Red boxes highlight the gene, and the peak associated with it. The point of the red arrow shows the position of the TSS; arrow direction indicates orientation of the gene. (B) ChIP-qPCR data for *FaADH*, *FaHY5*, and *FaTRAB1* showing percentage input over the two time points (mean ±SD, *n*=3). (C) Expression of FaMADS1 during fruit storage; real-time qPCR and RNAseq log_2_-fold change in red (mean ±SD, *n*=3). (D) Total anthocyanin content of strawberries at harvest (H), and following storage for 5 d at 20 °C or 8 °C for 5 d (mean ±SD, *n*=3); different letters indicate significant differences (*P*<0.05) based on a Dunn test.

Of these, three were used to validate the ChIP-seq data: alcohol dehydrogenase (ADH)-like (Fxa2Ag101499), HY5 (Fxa2Ag102914), and TRAB1 (Fxa2Dg200568). ChIP-seq IGV tracks aligned to the RNA-seq tracks (Fig. 4A) show a ChIP peak close to the promoter region of each of the three genes at the TSS or just before it. ChIP-qPCR for these three genes showed the expected repressive trend associated with H3K27me3 modification, with the day 5 sample being more enriched than the day 0 sample, although the difference was not significant, perhaps due to the large variation between the biological replicates (Fig. 4B).

*FaTRAB1* negatively regulates *FaMADS1* (Fxa6Cg103935) during fruit ripening (Lu *et al.*, 2023). *FaMADS1* was up-regulated during fruit storage between day 0 and day 5 (Fig. 4C) although the change was not significant, probably due to high variability in the day 0 expression across replicates. Downstream of TRAB1 and HY5 are genes related to the biosynthesis of anthocyanins (Lu *et al.*, 2023; Li *et al*., 2020; Liu *et al.*, 2023). The effect of chilled post-harvest storage at 8 °C in the dark on total anthocyanins was therefore compared with storage at 20 °C in the light (Fig. 4D). There was a significant increase in anthocyanin content when fruit were stored at 20 °C in the light, whereas there was no significant increase when stored at 8 °C in the dark (*P*<0.05).

## Discussion

An analysis of the chromatin profile of strawberry fruit during chilled post-harvest storage indicated that there was an association between several thousand genes expressed during post-harvest storage and the H3K27me3 mark.

The position of the H3K27me3 peaks relative to the gene differs from that seen in other plant systems such as peach buds (de la Fuente *et al*., 2015), Arabidopsis seedlings (Zhang *et al*., 2007), and *Brassica rapa* leaves and inflorescences (Payá-Milans *et al*., 2019) where the peaks are distributed evenly across the gene body. Here we detected a bias toward the 5' end of the gene, with peaks concentrated largely within the first 1000 bp of the promoter around the TSS, decreasing across the gene body towards the 3' end. This unusual distribution of the H3K27me3 mark is in agreement with its distribution on two expansin genes previously described in *F. vesca* (Mu *et al.*, 2021), suggesting a non-canonical distribution of H3K27me3 marks on strawberry chromatin. The H3K27me3 pattern observed here does not appear to be related to the tissue type, as the more canonical distribution of H3K27me3 has been described for another soft fruit, tomato (Cigliano *et al.*, 2022). Neither does this pattern appear to be a feature of the *Rosaceae* in general since in peach buds the H3K27me3 distribution was as expected (de la Fuente *et al.*, 2015). Indeed, the H3K27me3 pattern in strawberry is more reminiscent of the distribution of the H3K4me3 mark, a chromatin modification generally associated with a positive regulation of gene expression (Foroozani *et al.*, 2021). The median length of the H3K27me3 marked regions (987 bp) was more similar to that found in peach buds and Arabidopsis seedlings (Zhang *et al.*, 2007; de la Fuente *et al.*, 2015) while in the *B. rapa* study (Payá-Milans *et al.*, 2019) the marked region was >2 kb. This indicates that the H3K27me3 deposition mechanism may be different in strawberry fruit compared with other plant species and tissues.

The 39% of genes associated with H3K27me3 modification (and 12% of the genome) found here in chilled stored mature strawberry fruit is high compared with some other studies, with ~27% in *B. rapa* leaves and inflorescences, ~17% in Arabidopsis seedlings (Zhang *et al.*, 2007; Payá-Milans *et al.*, 2019), and 6.2% of the genome in tomatoes stored at room temperature (Cigliano *et al.*, 2022). However, H3K27me3 is detected on as many as 60% of protein-coding genes in the Arabidopsis shoot apical meristem (You *et al.*, 2017), indicating that the proportion of silenced genes can vary widely across species and tissues. H3K27me3 has to date not been assessed for its role in regulating genes in mature strawberry fruit, hence a high number of H3K27me3 marks is possible in this highly differentiated tissue. Given the repressive nature of the H3K27me3 mark, this would suggest that it is involved in wide-scale repression of gene expression in mature strawberry fruit.

Although 440 genes were identified whose expression pattern is consistent with H3K27me3-mediated gene repression, a large number of the H3K27me3-associated genes did not show this pattern. Indeed >20 000 genes at each time point were expressed but associated with the repressive H3K27me3 mark. Note that, for example, bivalent domains have been discovered where chromatin is modified in both an active and a repressive way simultaneously (Bernstein *et al.*, 2006; Faivre and Schubert, 2023). Indeed, H3K27me3 does not always prevent gene transcription and the independent accumulation of the active H3K4me3 mark (Liu *et al.*, 2014). For example, in potato, cold stress increased chromatin accessibility at bivalently H3K4me3- and H3K27me3-marked active genes (Zeng *et al.*, 2019).

The presence of bacterial DNA in the ChIP-seq dataset is consistent with an increase in microflora development over the 5 d of storage noted in other post-harvest studies (e.g. Wang *et al.*, 2018). Of the 440 genes whose expression pattern is consistent with repression by H3K27me3, a total of 16 were annotated as responding to biotic stimuli. This raises the possibility that H3K27me3 may be regulating responses to the increase in microflora associated with post-harvest storage.

The number of ChIP peaks increased at day 5 of storage compared with day 0, indicating that the role of this repressive mark is increasing. In comparison, a post-harvest study in tomato showed a reduction of genes associated with the H3K27me3 mark during storage (Cigliano *et al.*, 2022) and it was suggested that H3K27me3 loss could be responsible for activating genes required for fruit softening and senescence. However, in Cigliano *et al.* (2022) the fruit was not chilled during storage, suggesting that the increase in H3K27me3-associated genes may be influenced by the storage conditions.

GO term enrichment indicated that the most significant group of H3K27me3-regulated genes whose expression changes during storage are involved in abiotic stress responses, and the majority of these are down-regulated. This is perhaps unexpected given that cold storage is likely to be imposing abiotic stress. Down-regulation of abiotic stress-responsive genes was previously recorded for halved strawberries between day 1 and 5 of storage at 8 °C (Baldwin *et al.*, 2023) and may be due to effects of post-harvest senescence on the ability of the fruit to respond to the stress.

GO term enrichment also suggested that cold stress genes may be regulated by H3K27me3 in the stored fruit, although again expression of the majority of these genes was down-regulated with fruit storage. This contrasts with a fall in the H3K27me3 mark associated with the activation of stress-responsive transcription factors in cold-stressed rice seedlings (Dasgupta *et al.*, 2022). In Arabidopsis seedlings, cold stress caused a permanent decrease of H3K27me3 association in some cold-responsive genes (Kwon *et al.*, 2009). Thus, association with H3K27me3 is cold sensitive, and may cause permanent alterations in the epigenetic state. However, here the effect of cold storage seems to increase gene repression via H3K27me3 rather than alleviate it.

The integration of RNA-seq and ChIP-seq data also suggested a role for H3K27me3 in the regulation of cell wall breakdown. This is consistent with the loss of H3K27me3 modification associated with *FveEXP33* expression during *F. vesca* fruit ripening (Mu *et al.*, 2021) as expansin proteins participate in cell wall loosening and therefore play a role in fruit ripening and softening (Moya-León *et al.*, 2019). Here, a close match to *FveEXP33*, Fxa7Bg202431, was included in the 440 genes likely to be H3K27me3 regulated, but was down-regulated during fruit storage. Indeed, the majority of the cell wall-associated genes here associated with H3K27me3 changes were down-regulated, suggesting that during storage there is a repression of cell wall modulation, perhaps as a result of a cold-induced lowering of the metabolic rate.

Strawberry fruit are non-climacteric (Gu *et al.*, 2019), and ripening after harvest is limited, but does include increases in anthocyanins (Chen *et al.*, 2014). Chilled storage delays the post-harvest ripening process in strawberries and is used in the supply chain to maintain quality. It was shown here to abolish the increase in anthocyanins seen during post-harvest ripening at ambient temperature. One of the genes used to validate the ChIP-seq, *FaHY5* (Fxa2Ag102914), is light induced, and in *F. vesca FvHy5* regulates anthocyanin biosynthesis (Liu *et al.*, 2023) via a complex with FvbHLH9. Moreover, in *F. ananassa*, a B-box transcription factor, FaBBX22, interacts with FaHY5 to promote anthocyanin accumulation in strawberry fruits (Liu *et al.*, 2022). The finding here that *FaHY5* is repressed during fruit storage and is associated with the H3K27me3 mark provides a further potential mechanism for the regulation of anthocyanin biosynthesis in strawberry fruit. Its repression may be part of the shutting down of fruit ripening by the combination of the cold and dark storage conditions generally used in the supply chain and applied here.

The confirmation of ADH as another gene repressed during fruit storage, and its association with the H3K27me3 mark during storage of strawberry fruit is of interest as ADH is a key enzyme in ester biosynthesis, and esters are major components of strawberry aroma (Yan *et al.*, 2018). Chilled storage has significant effects on the volatile organic compound profile of strawberries (Baldwin *et al.*, 2023), including a reduction in the relative abundance of esters, especially non-acetate esters. The final enzyme in ester biosynthesis AAT was also down-regulated during strawberry fruit storage (Baldwin *et al.*, 2023) but was not associated with a H3K27me3 ChIP-seq peak, indicating that different mechanisms operate to modulate different steps of ester biosynthesis during fruit storage.

**Fig. 5.**
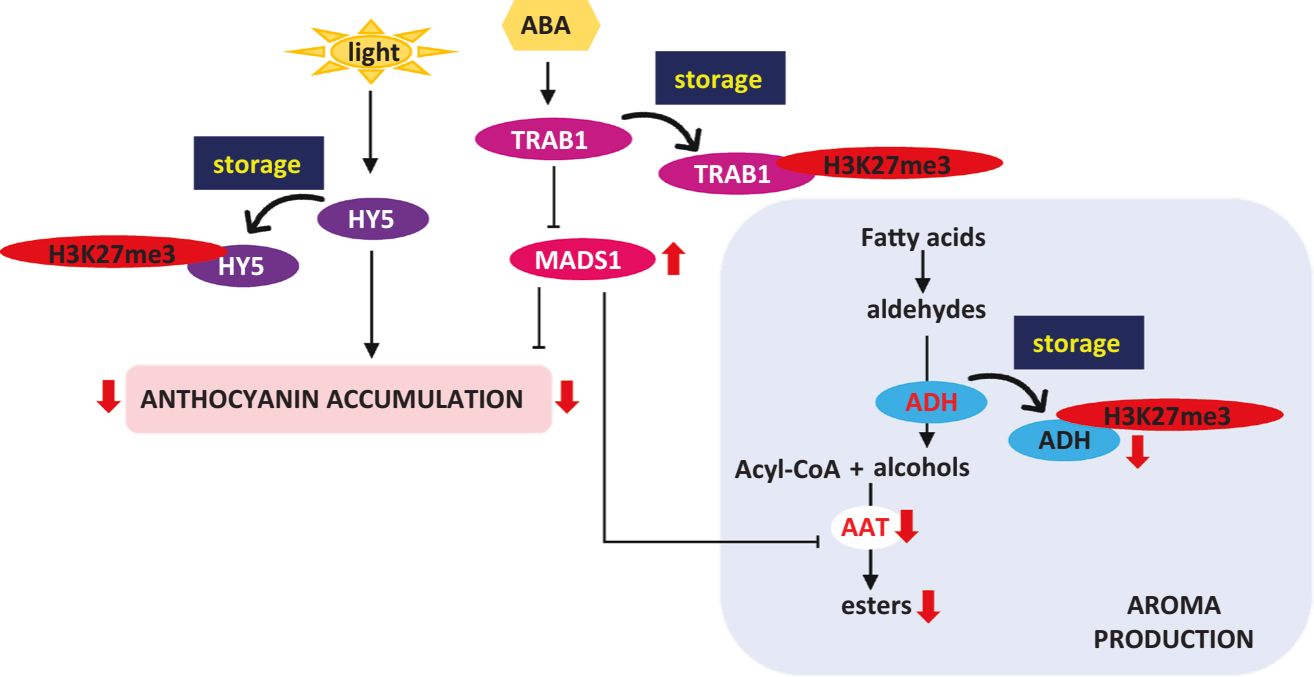
Model showing H3K27me3 regulation of fruit metabolism during storage. Cold dark storage of ripe strawberry fruit results in repression of *FaHY5* transcription through the H3K27me3 mark. This reduces anthocyanin accumulation. Storage also represses FaTRAB1 through the H3K27me3 mark, resulting in increased FaMADS1 expression which allows it to repress anthocyanin biosynthesis as well. An increase in MADS1 expression also represses AAT, resulting in reduced ester production. ADH is also regulated by the H3K27me3 mark during storage, resulting in reduced substrate for AAT, thus also contributing to repression of aroma development.

*FaTRAB1* is also an important gene in strawberry fruit ripening and aroma development, as it represses the expression of *FaMADS1* through an ABA-dependent mechanism (Lu *et al.*, 2023). In turn, FaMADS1 represses a number of ripening-related genes including AAT. The H3K27me3-dependent down-regulation of *FaTRAB1* during cold storage would therefore be expected to up-regulate *FaMADS1* expression, which indeed it does. The role of these genes can be summarized in a model (Fig. 5). The model predicts that during dark chilled storage, HY5, TRAB1, and ADH expression is subject to H3K27me3-dependent down-regulation. This leads to a reduction of anthocyanin and ester biosynthesis through two linked routes. Repression of the activator HY5 and the de-repression of the repressor MADS1 results in the reduction of anthocyanin biosynthesis, and de-repression of MADS1 and repression of ADH results in the reduction of ester formation. Our model is supported by (i) a lack of anthocyanin accumulation and a reduction in ester production during chilled dark storage; (ii) the association of H3K27me3 marks with HY5, TRAB1, and ADH during chilled dark storage; and (iii) the reduced expression of HY5, TRAB1, and ADH, and increased expression of MADS1 during chilled dark storage.

In conclusion, the exploration of the effects of H3K27me3 regulation on gene expression during strawberry fruit storage reveals mechanisms for the repression of key genes associated with strawberry ripening. This provides additional mechanisms for regulating aroma production post-harvest and potential targets for improving aroma retention in the supply chain.

## Supplementary data

The following supplementary data are available at *JXB* online.

Fig. S1. Visualization of the ChIP-seq reads in the three replicates.

Fig. S2. Efficiency of the H3K27me3 pull-down.

Fig. S3. ChIP-seq replicate GC analysis.

Fig. S4. Analysis of RNA-seq data.

Fig. S5. Comparison of RNA-seq and ChIP-seq data.

Table S1. ChIP-seq QC statistics.

Table S2. All PCR primers used.

Table S3. Mapped reads for ChIP-seq samples.

Table S4. Mapping statistics of RNA-seq reads.

Table S5. Significant KEGG pathways generated from DEGs.

Table S6. GO term annotations for RNA-seq genes associated with ChIP-seq peaks.

Table S7. Functional annotation of 440 genes whose expression pattern and ChIP-peak association is consistent with H3K27me3 regulation.

erae464_suppl_Supplementary_Figures_S1-S5_Tables_S1-S5

erae464_suppl_Supplementary_Table_S6

erae464_suppl_Supplementary_Table_S7

## Data Availability

Sequencing data are deposited in the NCBI Sequence Read Archive (http://www.ncbi.nlm.nih.gov/Traces/sra) under BioProject ID: PRJNA1096352.
