# Supplementary material for: The H3K27me3 histone mark correlates with repression of colour and aroma development post-harvest in strawberry fruit
Source: J Exp Bot. 2024 Nov 15;76(9):2487–99. doi: 10.1093/jxb/erae464 (PMC12187346; doi:10.1093/jxb/erae464)
Supplement: erae464_suppl_Supplementary_Figures_S1-S5_Tables_S1-S5 [file erae464_suppl_supplementary_figures_s1-s5_tables_s1-s5.pdf]

Baldwin et al. Supplementary Tables and Figures

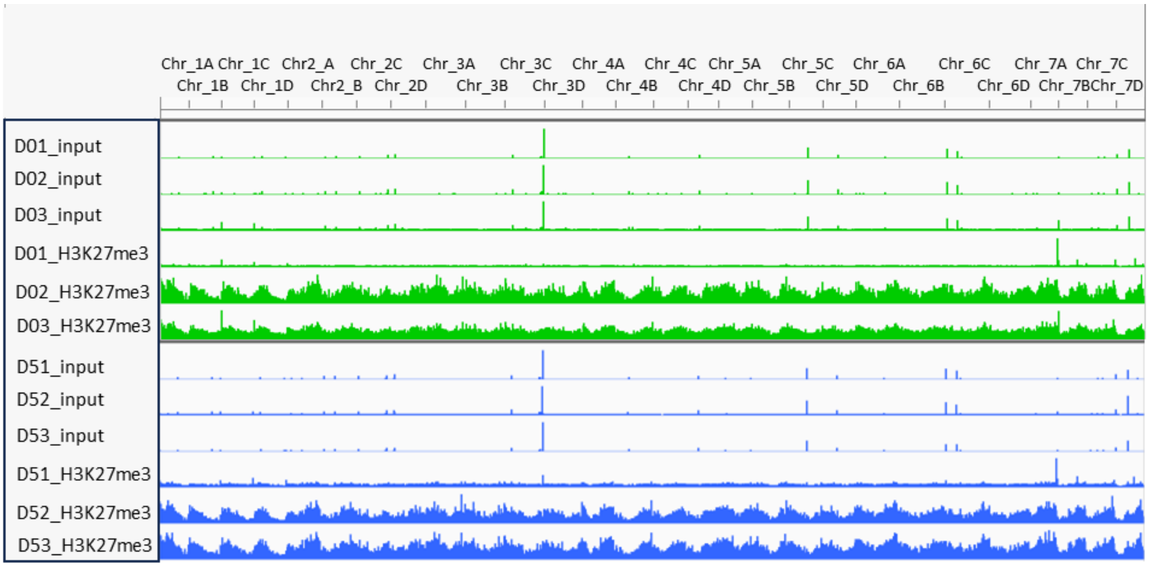

Supplementary Figure S1 IGV visualisation of the *Fragaria x ananassa* cv. Royal Royce chromosomes with the BigWig files from all day 0 (D0, green) and day 5 (D5, blue) samples. Peaks indicate positions in the genome where there is a higher ChIP-seq read density and therefore enrichment. IGV visualisation of the BigWig files showed that replicate 1 in both day 0 and 5 was of poorer quality with less variation being observed between the pull-down and the input.

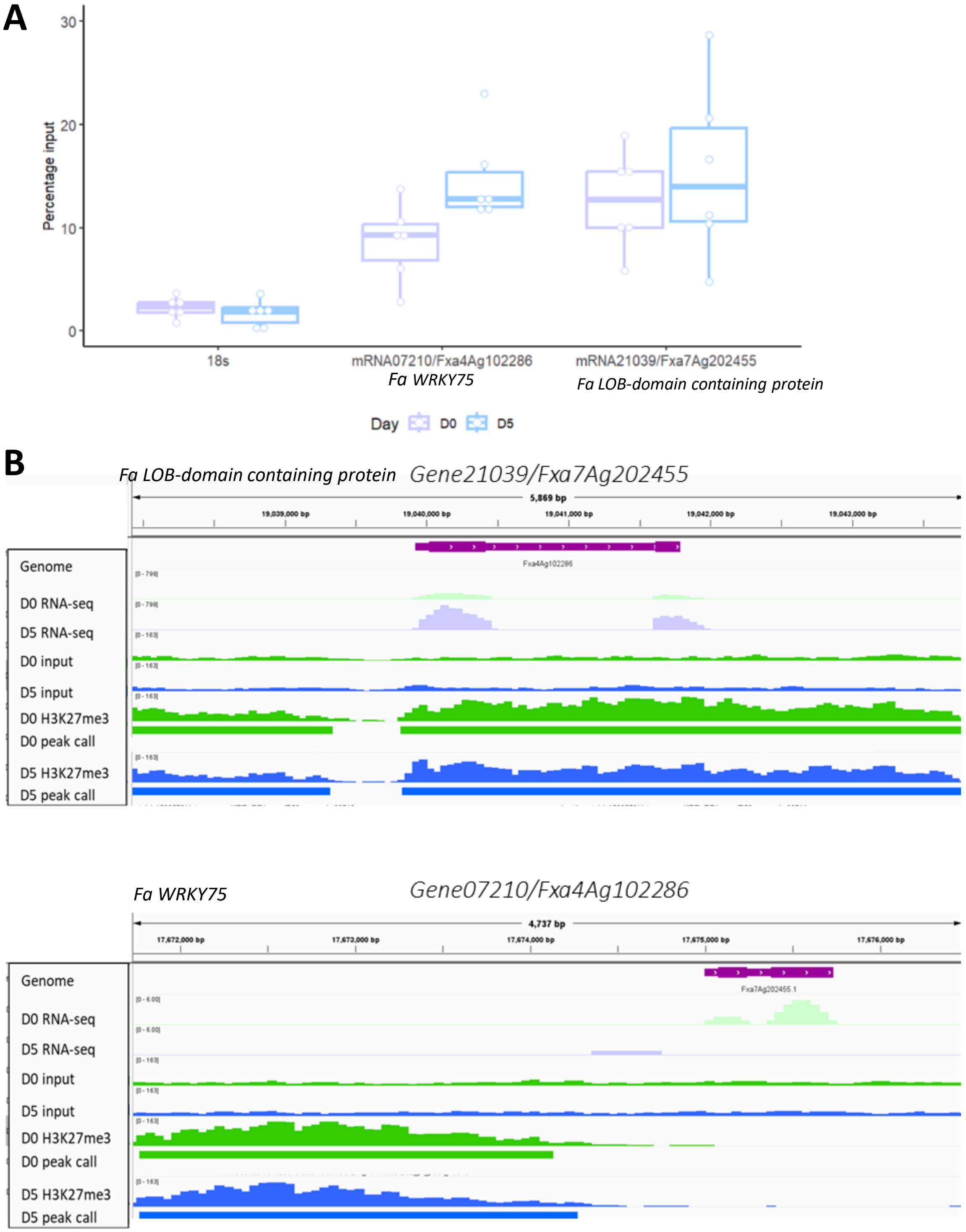

Supplementary Figure S2 (A) Efficiency of the H3K27me3 pull down shown by the % input of each gene Mean +/- SD, n=6 (three biological replicates with each biological replicate being represented by two separate pull-downs from two technical replicates). Both *Fragaria vesca* and *Fragaria x ananassa* cv. Royal Royce homologue gene codes are shown. **(B)** IGV tracks of RNA-seq data and ChIP-seq data of the two genes for day 0 (green) and day 5 (blue) of cold storage. Gene is in purple.

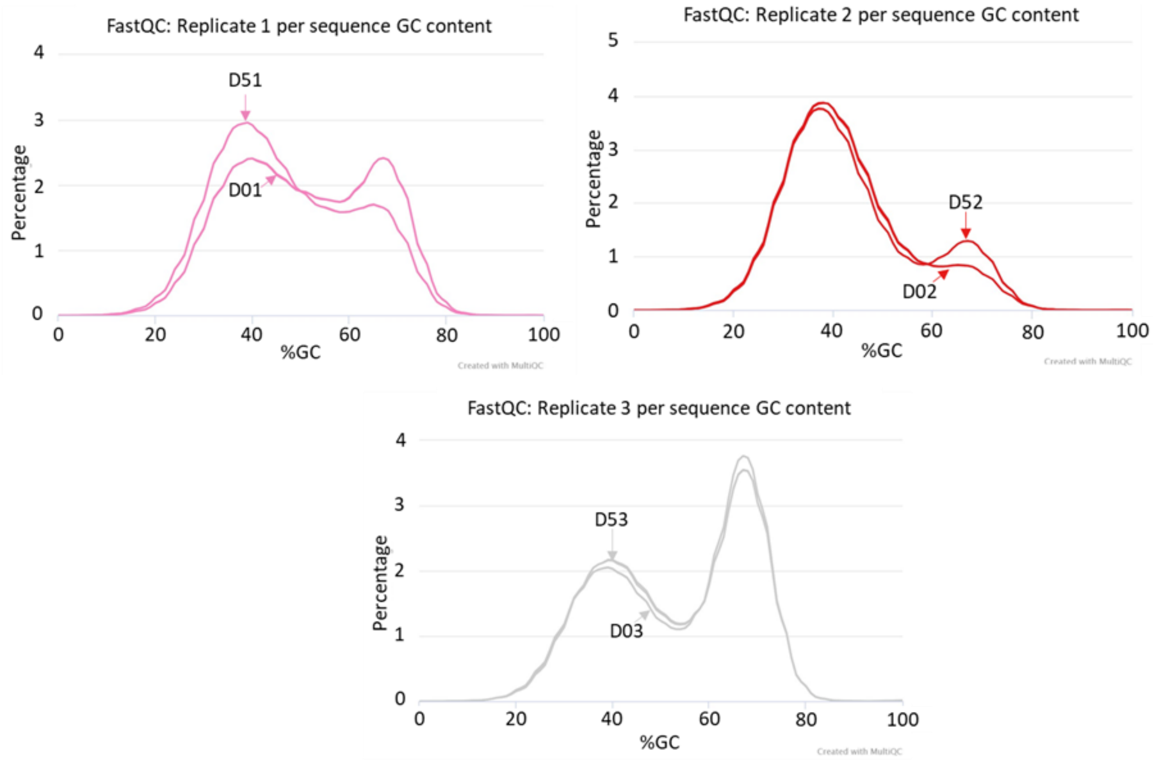

Supplementary Figure S3 ChIP-seq replicate GC analysis: % sequence GC content for each replicate. D0=day 0, D5=day 5, replicate numbers are 1,2 and 3

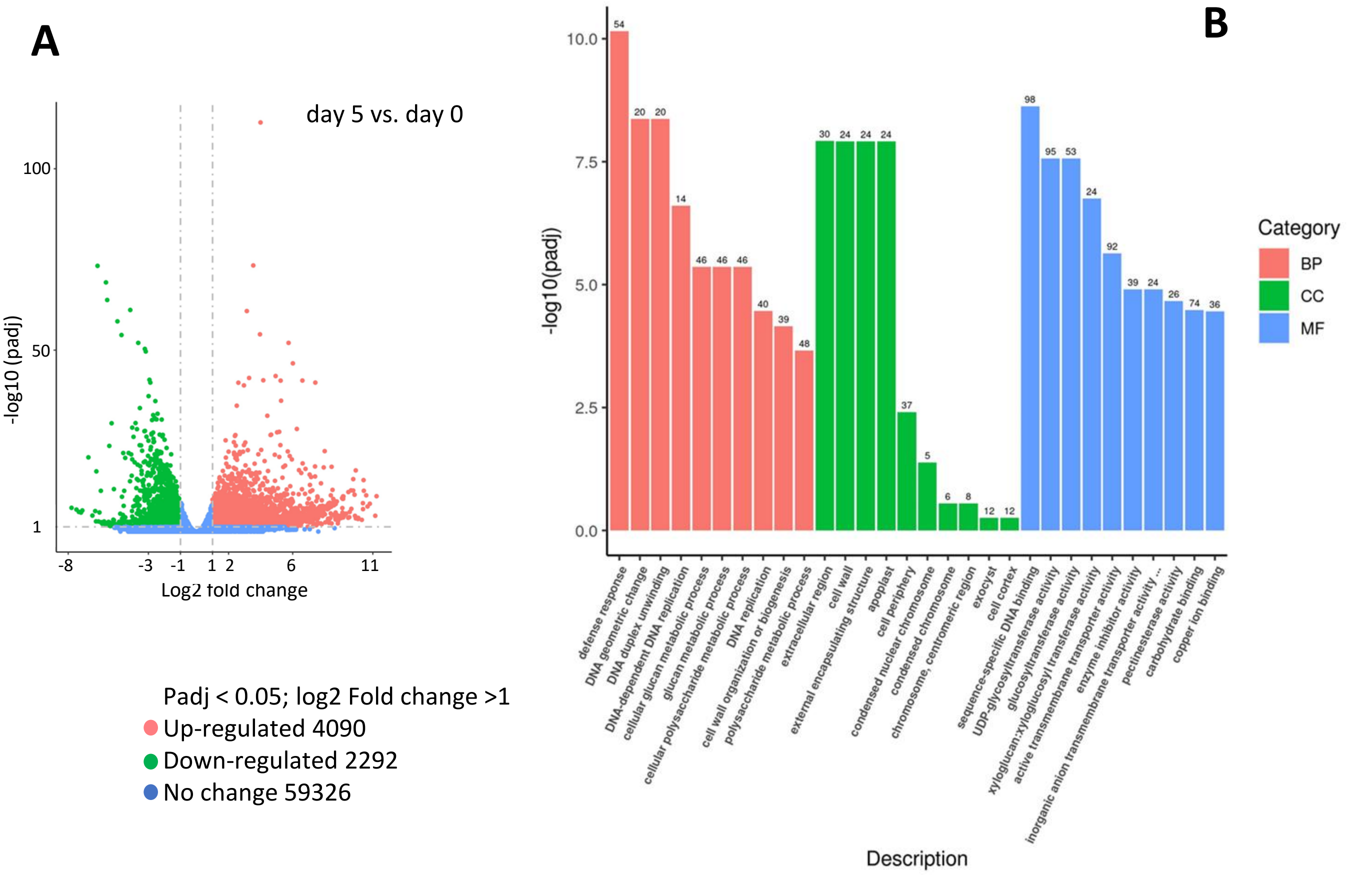

C

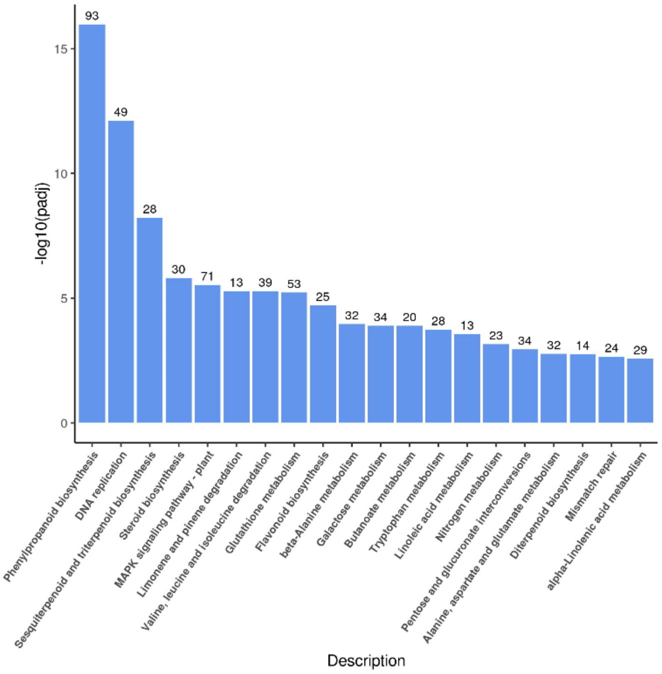

Supplementary Figure S4 Analysis of RNA-seq data (A) Volcano plot of DEGs between day 0 and day 5 of cold stored strawberry fruit. Genes highlighted in red and green represent the up and down-regulated DEGs respectively (padj<0.05, log2fc |>1) **(B)** Gene ontology (GO) term enrichment showing the top ten enriched terms for biological process (BP, red), cellular component (CC, green) and molecular function (MF, blue) for the wounded fruit across five days of cold storage. **(C)** KEGG enriched pathways for the strawberry fruit between day 0 (harvest) and day 5 of cold storage

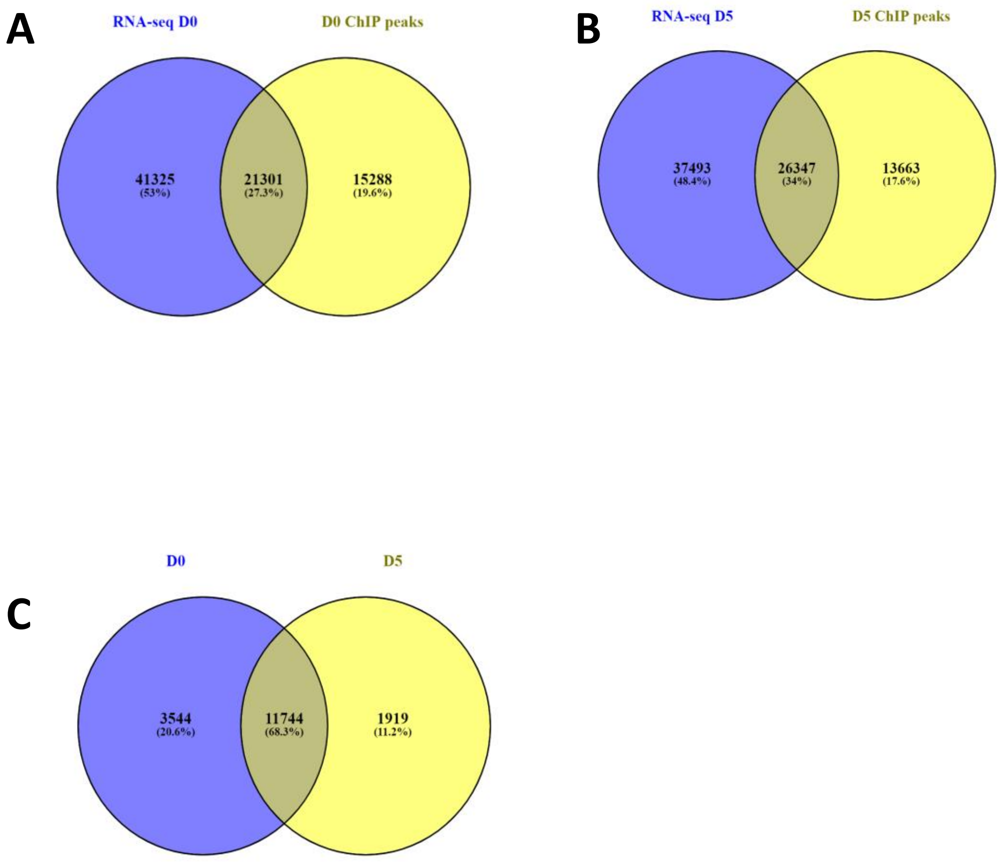

Supplementary Figure S5 Venn diagrams comparing RNA-seq and ChIP-seq data showing overlaps between (A) all genes expressed at day 0 and ChIP peaks at day 0 **(B)** all genes expressed at day 5 and ChIP peaks at day 5 **(C)** day 0 and day 5 ChIP peaks not associated with expressed genes at those timepoints.

Supplementary Table S1 ChIP-seq QC statistics for **(A)** the three biological replicates **(B)** two pseudo-replicates created from two biological replicates after removal of replicate 1 data. Fragment length (FragL), Relative cross-correlation coefficient (RelCC), squared sum of deviations (SSD) and percentage of reads in peaks (RiP%); (D0=day 0, at harvest, and D5 = day 5 of chilled storage; numbers refer to replicates).

A

Sample	Reads	FragL	RelCC	SSD	RiP%
**D01_H3K27me3**	647045	153	2.01	4.93	46.6
**D02_H3K27me3**	1824026	300	1.24	12.5	79.6
**D03_H3K27me3**	742994	161	1.4	5.68	62
**D51_H3K27me3**	1059884	157	1.48	7.27	61.3
**D52_H3K27me3**	1436650	300	1.35	10.8	78
**D53_H3K27me3**	692170	300	1.32	7.05	74.2

B

Sample	Reads	FragL	RelCC	SSD	RiP%
**D02_H3K27me3**	1283333	300	1.25	9.56	68.2
**D03_H3K27me3**	1283687	300	1.24	9.55	68.1
**D52_H3K27me3**	1064729	300	1.31	9.1	70.7
**D53_H3K27me3**	1064091	300	1.33	9.09	70.7

Supplementary Table S2 All PCR primers used

Target	Application	Primer name	Sequence (5' – 3')	Tm (°C)	Fragment size (bp)
18s rRNA small subunit ribosomal protein	PCR	PUV2	TTCCATGCTAATGTATTCAGAG	55.10	459
		PUV4	ATGGTGGTGACGGGTGAC	61.20	
40s	qPCR	40S_F	GTTCGTACCCATCTGCGTAATA	58.03	129
		40S_R	CTCAGCAAGGTAGTGACTAATCA	57.67	
WRKY75	ChIP-qPCR	B4TSS_Gene07210/Fxa4Ag102286_F	GCCCGCATAAAACTTCAGCT	59.19	102
		B4TSS_Gene07210/Fxa4Ag102286_R	ATCGATGGAGAGAGGAGTCG	58.11	
LOB domain-containing protein	ChIP-qPCR	B4TSS_Gene21039/Fxa7Ag202455_F	GGTCACGGGATCAGTCTTCA	59.1	116
		B4TSS_Gene21039/Fxa7Ag202455_R	AAGCAAAACAAGCAGGCAGA	58.89	
18s	ChIP-qPCR	B418SF	TGGCGACGCATCATTCAAAT	58.91	190
		B418SR	ATTGTCACTACCTCCCCGTG	59.1	
Ethylene-responsive transcription factor RAP2-10	qPCR	Fxa1Cg101455_F	TCGATACGGAAGAAGGCCAC	59.54	158
		Fxa1Cg101455_R	CAACTAATTTCCTCCGGGTCG	58.72	
Alcohol dehydrogenase-like	qPCR	Fxa2Ag101499_F	GGGGTTGCTGTACTTGTTGG	59.4	144
		Fxa2Ag101499_R	CTTCTCCACAACACCAGGGA	59.24	
Transcription factor HY5	qPCR	Fxa2Ag102914_F	AGCAAGGGAGAGGAAGAAGG	58.72	153
		Fxa2Ag102914_R	CGGCTTGCTGTTGTGTTCTT	59.62	
Probable pectate lyase 20	qPCR	Fxa2Bg201844_F	CCATCAACAGTCAGGGCAAC	59.12	108
		Fxa2Bg201844_R	ATCTCCAGTTCCAGTGCCTC	59.09	
bZIP transcription factor TRAB1	qPCR	Fxa2Dg200568_F	AGCTCGTAAACAGGCCTACA	58.74	168
		Fxa2Dg200568_R	CGTCTCAAGCACTGCCTTTT	59.05	
Fatty acid 2-hydroxylase 1-like	qPCR	Fxa3Ag101008_F	GCACCATGGGAAGCCGTC	61.13	127
		Fxa3Ag101008_R	TGTTCCGAAAACATTGTCCCA	58.34	
Granule-bound starch synthase 2	qPCR	Fxa6Ag104600_F	CTTGGGTGGACGTTTGACAG	59.06	148
		Fxa6Ag104600_R	GAGCAGCTTTGTCCCAACTT	58.68	
Probable chalcone--flavonone isomerase 3	qPCR	Fxa7Dg102232_F	TCCAGCTACTTCCCCAACAG	59.02	134
		Fxa7Dg102232_R	GAGACACCCCTCTTGTTCCA	58.94	
Alcohol dehydrogenase-like	ChIP-qPCR	Fxa2Ag101499_TSS_F	ATGGATTGTAGCTGCGGTTG	58.62	116
		Fxa2Ag101499_TSS_R	ACAGGGCAGTGAAGAGGATC	59.09	
Transcription factor HY5	ChIP-qPCR	Fxa2Ag102914_TSS_F	GCCCTCCTCCCTTGTTTACT	59.01	185
		Fxa2Ag102914_TSS_R	GAAGACCTCTCACTGCTGGA	58.74	
bZIP transcription factor TRAB1	ChIP-qPCR	Fxa2Dg200568_TSS_F	GCTTGCCTAGTCAGCTGTTG	59.2	103
		Fxa2Dg200568_TSS_R	TCCCACCATTTACATCCCCG	59.45	
Mads1	qPCR	Fxa6Cg103935_F	AACATGCTCAGACACAGGGA	63.5	252
		Fxa6Cg103935_R	ATTGACTGGTTGAGCATGGG	64.90	

Supplementary Table S3. Bowtie mapped reads for ChIP samples RR = cv. Royal Royce and Cam =cv. Camarosa.

Day	Sample	Total reads	*Fragaria vesca mapped reads*	Average	*Fragaria ananassa cv. RR mapped reads*	Average	*Fragaria ananassa cv. Cam mapped reads*	Average
Day 0	**Input_1**	49967870	56.28%	57.22%	92.34%	89.01%	86.82%	85.37%
	**Input_2**	67053584	60.19%		92.78%		89.09%	
	**Input_3**	77704802	55.20%		81.92%		80.20%	
	**H3K27me3_1**	46036568	30.50%	37.60%	45.66%	53.72%	45.18%	53.25%
	**H3K27me3_2**	68913946	55.14%		76.41%		75.81%	
	**H3K27me3_3**	56678518	27.15%		39.10%		38.76%	
Day 5	**Input_1**	58552768	57.66%	58.64%	93.96%	90.46%	88.43%	86.74%
	**Input_2**	75139322	63.20%		93.81%		91.15%	
	**Input_3**	59269046	55.07%		83.61%		80.64%	
	**H3K27me3_1**	59110544	38.74%	40.03%	56.47%	55.64%	55.87%	55.16%
	**H3K27me3_2**	52819858	56.76%		76.71%		76.15%	
	**H3K27me3_3**	58344552	24.60%		33.74%		33.47%	

Supplementary Table S4 Mapping statistics of RNA-seq reads (D0=day 0, at harvest, and D5 = day 5 of chilled storage; numbers refer to replicates).

sample	total_reads	total_map	unique_map	multi_map	proper_map
D01	84898946	78555776(92.53%)	69864314(82.29%)	8691462(10.24%)	64569464(76.05%)
D02	91459644	85930545(93.95%)	76248134(83.37%)	9682411(10.59%)	70773252(77.38%)
D03	81949700	76911619(93.85%)	67837408(82.78%)	9074211(11.07%)	62444798(76.2%)
D51	83444000	78355820(93.9%)	69319669(83.07%)	9036151(10.83%)	64449054(77.24%)
D52	86324616	80819937(93.62%)	71301008(82.6%)	9518929(11.03%)	66265084(76.76%)
D53	88966212	84216636(94.66%)	74470781(83.71%)	9745855(10.95%)	69446364(78.06%)

Supplementary Table S5 Significant KEGG pathways generated from DEGs (padj<0.05, log2fc |>1) between strawberry fruit at harvest (day 0) and following 5 days of chilled storage (day 5)

KEGGID	Description	pvalue	padj
fve00940	Phenylpropanoid biosynthesis	8.88E-19	1.05E-16
fve03030	DNA replication	1.31E-14	7.70E-13
fve00909	Sesquiterpenoid and triterpenoid biosynthesis	1.50E-10	5.90E-09
fve00100	Steroid biosynthesis	5.31E-08	1.57E-06
fve04016	MAPK signaling pathway - plant	1.26E-07	2.97E-06
fve00903	Limonene and pinene degradation	2.74E-07	5.29E-06
fve00280	Valine, leucine and isoleucine degradation	3.14E-07	5.29E-06
fve00480	Glutathione metabolism	3.98E-07	5.87E-06
fve00941	Flavonoid biosynthesis	1.46E-06	1.92E-05
fve00410	beta-Alanine metabolism	9.16E-06	0.000108
fve00052	Galactose metabolism	1.19E-05	0.000126
fve00650	Butanoate metabolism	1.28E-05	0.000126
fve00380	Tryptophan metabolism	1.99E-05	0.00018
fve00591	Linoleic acid metabolism	3.29E-05	0.000277
fve00910	Nitrogen metabolism	8.73E-05	0.000687
fve00040	Pentose and glucuronate interconversions	0.000152	0.001118
fve00250	Alanine, aspartate and glutamate metabolism	0.000247	0.001712
fve00904	Diterpenoid biosynthesis	0.000269	0.001766
fve03430	Mismatch repair	0.000357	0.002215
fve00592	alpha-Linolenic acid metabolism	0.000458	0.002704
fve00905	Brassinosteroid biosynthesis	0.000638	0.003583
fve04933	AGE-RAGE signaling pathway in diabetic complications	0.001414	0.007583
fve00460	Cyanoamino acid metabolism	0.001563	0.00802
fve00900	Terpenoid backbone biosynthesis	0.001761	0.008659
fve00310	Lysine degradation	0.003452	0.016294
fve00340	Histidine metabolism	0.003658	0.016602
fve00620	Pyruvate metabolism	0.00387	0.016912
fve00966	Glucosinolate biosynthesis	0.006133	0.025695
fve00500	Starch and sucrose metabolism	0.006525	0.025695
fve00053	Ascorbate and aldarate metabolism	0.006533	0.025695
fve00430	Taurine and hypotaurine metabolism	0.0073	0.027788
fve00640	Propanoate metabolism	0.009023	0.033273
fve00072	Synthesis and degradation of ketone bodies	0.011059	0.039544
fve00565	Ether lipid metabolism	0.011851	0.041131
